# Human immunodeficiency virus type 1 (HIV-1) proviral DNA load in purified CD4+ cells by LightCycler^® ^Real-time PCR

**DOI:** 10.1186/1471-2334-5-15

**Published:** 2005-03-21

**Authors:** Benoît Kabamba-Mukadi, Philippe Henrivaux, Jean Ruelle, Nicole Delferrière, Monique Bodéus, Patrick Goubau

**Affiliations:** 1 Laboratoire de référence SIDA, Université Catholique de Louvain, Avenue Hippocrate 54, B1200 Bruxelles, Belgium; 2 Service de Médecine interne, Clinique Saint Joseph, Liège, Belgium

## Abstract

**Background:**

The human immunodeficiency virus type 1 (HIV-1) proviral DNA persists in infected cells, even after prolonged successful HAART. In the present study, a relative quantification assay of HIV-1 proviral DNA by LightCycler^® ^real-time PCR based on SYBR Green I detection was developed in comparison to the number of purified CD4+ cells as estimated by the quantification of the β-globin gene.

**Methods:**

The ability of the designed *gag *primers to quantify HIV-1 Group M and the PCR efficiency were assessed on HIV-1 reference isolate subtypes A, B, C and D. The 8E5 cell line containing a single defective copy of HIV-1 proviral DNA was used as a standard for both the HIV-1 target gene and the β-globin reference gene. The assay was applied on thirty consecutive patient samples received for RNA viral load determinations and on retrospective samples from fifteen patients undergoing 2 years of structured treatment interruption (STI).

**Results:**

The lower limit of quantification was 50 HIV-1 DNA proviral copies per CD4+ cell sample. The dynamic range was from 50 to 10^6 ^HIV-1 DNA copies per CD4+ cell sample with intra- and inter-assay coefficients of variability ranging from 3.1% to 37.1%. The β-globin reference gene was quantified down to a limit of 1.5 pg of DNA/μl (approximately 5 cells) with intra- and interassay coefficients of variability ranging from 1.8% to 21%. DNA proviral load varies widely among HIV-1 infected patients. Proviral load and plasma viral load rebound were high in STI patients who took longer to achieve an undetectable plasma viral load under therapy. A statistically significant correlation was observed between DNA proviral load and RNA steady state viral load in STI patients (p-value = 0,012).

**Conclusion:**

We have developed a fast, sensitive and specific relative quantification assay of HIV-1 proviral DNA in purified CD4+ cells. The assay enables the monitoring of HIV-1 proviral load, which may be useful to monitor therapy efficacy especially in patients with undetectable plasma RNA viral load, and allows the exploration of viral reservoirs.

## Background

At present, HIV-1 infected patients are followed by monitoring plasma HIV-1 RNA viral load, allowing follow up of the immediate effects of treatment, and CD4+ T cell count, allowing initiation or reinitiation of the therapy as immune function decreases [1]. The HIV-1 proviral DNA load could be an alternative viral marker, as it is known that proviral DNA persists in infected cells, even after prolonged successful HAART as evidenced by undetectable plasma RNA viral load. A decline in DNA might indicate a long-term impact of the HAART on the reservoirs and the long-term effectiveness of the treatment [2-11]. But data regarding the decline in DNA are sometimes conflicting. Some authors noted decreased levels after one year of antiretroviral triple combination therapy [12] and others reported stable HIV-1 DNA levels over several years in PI-ART naive patients [13,14]. Several assays for the quantification of HIV type 1 proviral DNA in peripheral blood mononuclear cells (PBMC) have previously been reported, and many of them are based either on the principle of conventional PCR requiring post PCR analysis or on real-time PCR on total PBMC, using specific probe detection [9,10,15-23]. Recently, a multiplex real-time PCR for quantification of HIV-1 DNA and the human albumin gene in CD4+ cells has been published [23] using TaqMan probes and an Epstein-Barr virus (EBV) standard curve. Sequence-specific hybridisation probes provide the most specific real-time analysis of amplified target sequences but the very high variability of HIV sequences within subtypes, even in HIV variants in a given patient, led us to choose a sequence-independent detection with SYBR Green I. One of the experimental prerequisites for relative quantification consists in having identical PCR efficiencies for both target gene and reference gene in sample, standard, and calibrator. PCR efficiency may vary between EBV standard PCR and HIV-1 PCR. We therefore used the 8E5 cell line containing a single defective copy of HIV-1 proviral DNA as a standard for both the HIV-1 target gene and the β-globin reference gene in order to carry out a relative quantification. Another recent publication described a real-time PCR based on LightCycler^® ^technology revealed through SYBR green fluorochrome to quantify the HIV-1 proviral DNA load[24]. The article describes quantification in PBMC of HIV-1 seropositive patients. As 95 to 99% of infected cells are CD4+ cells[25], we developed a relative quantification assay of HIV-1 proviral DNA in purified CD4+ cells on the LightCycler^® ^by real-time PCR compared to cell quantification by β-globin PCR.

## Methods

### Primer design and selection

Several primers were designed in the *gag-pol *junction region with low variability on the basis of a consensus sequence obtained by aligning complete sequences of HIV-1 subtypes A to J from the Entrez nucleotides database [26]. The Vector NTI^® ^Suite (InforMax, Bethesda, USA) software was used for the design of primers. Selection of primer sets was first based on a specific signal without primer dimer formation when testing DNA from 8E5 cell dilution series and negative controls. Primer and MgCl_2_ concentrations were optimised by combining primer concentrations ranging from 0.3 mM to 1.2 mM and MgCl_2 _concentrations from 2 mM to 5 mM. The selected primers were tested on DNA samples obtained from HIV-1 subtypes A, B, C, and D reference strains and from clinical samples including subtypes B and non-B. We also tested the absence of signal with HIV-2 DNA. The final primer set selection was based on the efficiency of the PCR reaction. Differences in efficiency may have a major impact on the calculation of the initial amount. In theory, the optimum efficiency in PCR is two, meaning that every PCR product is replicated once every cycle. In reality, however, many PCR parameters influence PCR efficiency which is calculated according to the formula E = 10^-1/slope^. The LightCycler^® ^Software calculates the slope of the standard curve by plotting crossing points against the logarithm of concentration for each standard point of a 10-fold dilution series. A slope value of -3.33 indicates the maximal PCR efficiency.

### Reference strains and patients

Four different reference strains of HIV-1 and 2 of HIV-2 from the National Institute for Biological Standards and Control (NIBSC, UK) were used in this project: HIV-1 Primary isolates-Uganda (HIV-1 subtype A, NIBSC repository reference ARP177.5) [27], 8E5/LAV (HIV-1 subtype B, NIBSC repository reference ARP110) [28], HIV-1 SE12808/ SE14784 (Subtype C, NIBSC repository reference ARP197.1, ARP197.2) and HIV1-ELI (HIV-1 subtype D, NIBSC repository reference EVA117) [29], HIV-2 ROD reference strain (subtype A, NIBSC repository reference EVA121.1) [30,31] and HIV-2 EHO reference strain (subtype B, NIBSC repository reference EVA132) [32].

Samples from 15 patients undergoing 2 years (start 2001) of structured treatment interruption (STI) and 30 consecutive clinical samples received for RNA plasma viral load determinations were included in the study. Informed consent following the Helsinki declaration was obtained from each patient. The HIV-1 seropositive status was confirmed according to the accepted methods. HIV-1 RNA plasma viral load was performed using the HIV-1 Amplicor™ Monitor 1.5 (Roche, Branchburg, NJ, USA). The viral RNA and the proviral DNA sequences were determined by using an in house RT-PCR method applied on reverse transcriptase and protease genes [adapted from [33]]. HIV RNA was extracted from plasma (QIAmp Viral Mini Kit™, QIAGEN, Leiden, The Netherlands) and a direct cycle sequencing was used with BigDye terminator chemistry on an ABI Prism 310 (Applied Biosystems, Foster City, USA).

CD4+ cells were isolated from 10 ml of patient EDTA whole blood samples by an immunomagnetic method using anti-CD4 coated magnetic beads (Dynabeads M450 CD4, Dynal A S, Oslo, Norway) according to the manufacturer's protocol and were stored at -80°C until use. The complete process takes approximately 3 hours and the purity of the CD4+ cell preparation was about 99% as estimated by Becton Dickinson Fascan Flow Cytometer technology (data not shown). HIV-2 clinical samples were obtained from patients who were diagnosed with HIV-2 infection at our AIDS reference laboratory. The 8E5 cell line, the CD4+ blood donor cells infected with HIV-1 reference strains, and the H9 cell line infected with HIV-2 reference strains were cultured in RPMI 1640 medium supplemented with 10% FetalClone 1^® ^(HyClone, Logan, Utah, USA), 1% Glutamine 200 mM and 0.1% Gentamycin 50 mg/ml. 8E5 cells were counted by the Coulter automated haematology analyser and diluted to 10^6 ^cells per aliquot stored at -80°C.

### DNA purification

DNA was extracted from purified patient CD4+ cells diluted in 200 μl PBS, using the High Pure^® ^PCR Template Preparation Kit (Roche Diagnostics GmbH, Mannheim, Germany) according to the manufacturer's recommendations. To concentrate the HIV-1 target gene in patient samples, DNA was eluted in a volume of 50μl. DNA from the T-lymphoblastoid 8E5 cells, and CD4+ blood donor cells infected with HIV-1 reference strains were eluted in 200μl. The 8E5 cell DNA was purified from 10^6 ^cells. A negative control (template replaced by Nuclease-free water) was included in each PCR run. Particular attention was paid to using DNAse and RNAse free materials. Depending on the number of samples, the whole DNA purification process required approximately 1 hour.

### HIV-1 DNA real time PCR assay

A series of ten-fold dilutions of 8E5 DNA corresponding to 2.5 × 10^3 ^to 2.5 HIV-1 DNA copies per μl (2.5 × 10^3 ^to 2.5 cells/μl) was included in each experiment in order to generate an external standard curve. The PCR mixture (total volume 20 μl) in nuclease free water contained 2 μl of LightCycler^® ^FastStart DNA Master SYBR Green 1, a ready-to-use "Hot Start" reaction mix (Roche Diagnostics GmbH, Mannheim, Germany), final concentrations of 4 mM MgCl_2 _and 0.5μM of each primer and 5μl of purified DNA or negative control. All samples were analysed in duplicate. The amplification protocol for HIV-1 on the LightCycler^® ^was as follows: a 10 min denaturation step at 95°C for polymerase activation, a "touch down" PCR step of 10 cycles consisting of 10 seconds (s) at 95°C, 10 s at 65°C, and 30 s at 72°C, followed by 40 cycles consisting of 10 s at 95°C, 10 s at 55°C, and 30 s at 72°C. The fluorescence was measured at the end of each elongation step. The next step was a slow heating (0.1°C per s) of the amplified product from 65°C to 95°C in order to generate a melting temperature curve. This curve served as a specificity control. The entire cycling process including data analysis took less than one hour and was monitored using the LightCycler^® ^software program (version 3.5). Second derivative maximum mode was chosen with baseline adjustment set in the arithmetic mode.

A fragment from the human β-globin gene was amplified in parallel with the HIV-1 *gag *gene to quantify the total number of investigated cellular genomes. The β-globin real-time quantitative PCR was performed using the LightCycler^® ^Control Kit DNA (Roche Diagnostics GmbH, Mannheim, Germany) according to the manufacturer's recommendations. The PCR mixture (total volume 20μl) in nuclease free water contained 2μl of LightCycler^® ^FastStart DNA Master SYBR Green 1 and 2μl of LightCycler^® ^Control Kit DNA primer mix, 2μl of 10-fold dilution of purified DNA or negative control, and a final concentration of 4 mM MgCl_2_. The DNA control from the kit corresponds to 30 ng of human genomic DNA. The same 8E5 DNA standard samples corresponding to 2.5 × 10^3 ^to 2.5 cells/μl were included in each experiment in order to generate an external standard curve as for the HIV-1 experiment. All samples were run in duplicate. A melting temperature step of β-globin amplicons was performed.

To validate the use of the 8E5 cells as standards, we first performed a real-time PCR quantification of the human β-globin gene using the DNA control of the kit to generate a standard curve. The values of the 8E5 cell count as performed by the Coulter automated haematology analyser were compared with the values obtained by extrapolating the cell count from the β-globin real-time PCR quantification. In the second step, 8E5 cells were used as standards and we compared the kit DNA control value obtained from the LightCycler^® ^quantification with the theoretical value.

Relative quantification was carried out using the LightCycler^® ^Relative quantification Software (version 1.0). The calculation of data is based on the crossing point (Cp) values obtained by the LightCycler^® ^Software. Results are calculated as the target/reference ratio of the sample divided by the target/reference ratio of the calibrator. This corrects for sample inhomogeneity and variability of detection. The result of the HIV-1 proviral quantification was expressed as log_10 _number of DNA copies per 10^6 ^CD4+ cells.

### Statistical analysis

Statistical analysis was performed using the parametric Pearson correlation test or the nonparametric Spearman correlation method. The critical *p*-value required to reject the null hypothesis (there is no proof of significant correlation between the variables) and accept the alternative hypothesis is equal to or less than 0,05.

## Results

### Primer selection

Out of several primers designed in the HIV-1 *gag-pol *junction region, the sequences of the selected HIV-1 *gag *forward primer BK1F and the reverse primer BK1R were respectively: 5'-GTA ATA CCC ATG TTT TCA GCA TTA TC-3' and 5'-TCT GGC CTG GTG CAA TAG G-3'. These amplify a 181-bp amplicon in the *gag *region of low variability among HIV-1 subtypes.

### Standard validation

**Figure 1:**
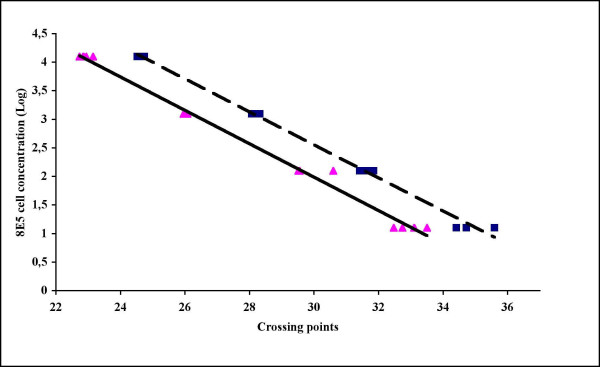
the HIV-1 and the human globin standard curves created by a ten-fold dilution series of 8E5 cell DNA show comparable slopes, indicating equal PCR efficiency. The LightCycler Software calculates the slope of the standard curve by plotting crossing points against the logarithm of concentration for each standard point. Each standard point was run in five replicates. (_____) HIV-1 DNA standard curve, (_ _ _) Human globin standard curve.

**Figure 2:**
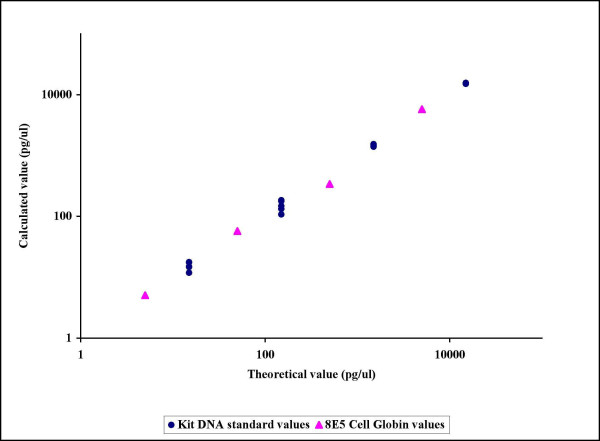
Validation of the use of 8E5 cells as standards for human globin PCR. The figure shows similar results of the 8E5 cell count as performed by the Coulter automated hematology analyzer compared with the values obtained by real-time PCR quantification of the human globin gene. The comparison of the theoretical and calculated values of the DNA control sample from LightCycler Kit shows equal results. Each standard point was run in five replicates.

The use of 8E5 cells as standards was validated. As shown in Figure 1, the HIV-1 and the β-globin curves generated by using 8E5 cells had comparable slopes, indicating equal PCR efficiency. The comparison of the 8E5 cell count as performed by the Coulter automated haematology analyser with the values obtained by real-time PCR quantification of β-globin genes showed similar results, as did the comparison of the DNA control sample value obtained from LightCycler^® ^quantification with the theoretical value (Figure 2).

### Specificity of the assay

As described in the methods section, the specificity of the PCR reaction was tested with DNA purified from the 8E5 cell line carrying a single copy of subtype B proviral DNA per cell, with CD4+ blood donor cells infected with HIV-1 subtype A, C, and D reference strains and with H9 cells infected with HIV-2 ROD (subtype A) and EHO (subtype B) reference strains. Clinical samples from HIV-1 or HIV-2 infected patients were included. The subtyping results based on proviral protease sequences showed 65% of subtype B viruses and 35% of non-subtype B viruses, which included subtypes A, C, D, F1 and CRF AE [34].

HIV-1 amplicons produced in the PCR reaction had the same melting temperature of 85.30°C, with a slight variation depending on the HIV-1 subtypes and on the type of initial sample, indicating that the signal was specific. The melting temperature of β-globin amplicons was about 86°C. Furthermore, the specificity of the HIV-1 amplification was confirmed by the presence of the expected band size on the electrophoresis gel (agarose 2%) carried out with the PCR products obtained from standards, reference strains, and patient samples. Direct sequencing of amplified products using the PCR primers showed the expected HIV-1 sequences (data not shown). No amplification was obtained with HIV-2 DNA purified from patient samples and from reference strains belonging to HIV-2 subtypes A and B. The four HIV-1 reference strains obtained from NIBSC were amplified. The results showed that the assay amplified DNA purified from HIV-1 reference isolate subtypes A, B, C, and D with similar PCR efficiency (Table 1).

**Table 1:** PCR efficiencies of HIV-1 group M amplification

**Sample concentration:**	**Subtype A**	**Subtype B**	**Subtype C**	**Subtype D**
Log dilution	Mean Cp^a^	Mean Cp	Mean Cp	Mean Cp
-2	26,16	22,90	24,61	26,13
-3	29,50	26,02	28,10	29,27
-4	32,82	29,75	31,79	32,86
-5	36,72	32,98	34,83	36,72
				
Slope^b^	-3,50	-3,37	-3,43	-3,54
R^2c^	0,99	0,99	0,99	0,99

(a) Mean of crossing point values calculated using two replicates of each point in 3 runs.

(b) The LightCycler Software calculates the slope of the curve by plotting crossing points against the logarithm of concentration for each point of a 10-fold dilution series. A slope value of -3.33 indicates the maximal PCR efficiency.

(c) The LightCycler Software also calculates the coefficient of correlation R^2^.

### Sensitivity and reproducibility of the assay

Ten replicates of 1 copy, 5 copies, and 10 copies of DNA purified from 8E5 cells were tested in the PCR reaction. The detection of one HIV-1 DNA copy per PCR failed in most cases while five and ten copies were always detected. The lower limit of quantification was set at 5 HIV-1 DNA proviral copies per PCR or 50 HIV-1 DNA proviral copies per CD4+ cell sample. A HIV-1 DNA proviral load below 50 copies per CD4+ cell sample was reported as undetectable. The dynamic range was from 50 to 10^6 ^HIV-1 DNA proviral copies per CD4 lymphocyte sample. We tested the intra and inter-assay reproducibility of our technique with a series of ten-fold dilutions of 8E5 DNA from 12.5 × 10^3 ^to 12.5 HIV-1 DNA copies per PCR. The intra-assay reproducibility was evaluated using five replicates of each point and the inter-assay reproducibility was calculated on 10 runs. The intra- and inter-assay coefficients of variability of the HIV-1 DNA copy number ranged from 3.1% for high provirus concentrations to 37.1% for low concentrations. The quantification of the human β-globin reference gene by using the "LightCycler^® ^Control Kit DNA" enabled a lower limit of detection of 1.5 pg/μl (approximately 5 cells) with intra- and interassay coefficients of variability ranging from 1.8% for high DNA concentrations to 21% for low DNA concentrations. We observed an inhibition of the human β-globin reference gene amplification at high amounts of the cellular genome. The inhibition was shown by a maximum fluorescence of the amplification curve, which was lower than the maximum fluorescence of the standard points. In our assay, this problem was solved by diluting (10-fold) DNA from patient samples. The influence of the type of sample on the PCR reaction was tested by diluting the standard 8E5 cells in HIV seronegative plasma from a blood donor before DNA extraction. A slight difference (about 1 Cp) was observed within the range of 12.5 to 12.5 × 10^3^HIV-1 DNA copies per PCR between the *Cp *values obtained for 8E5 cells diluted in PBS buffer as compared to 8E5 cells diluted in the plasma, in both HIV-1 and β-globin assays. We also successfully tested the quantification of both HIV-1 and β-globin genes without the need to include a standard curve in every run by loading an external standard curve generated in a different run.

### HIV-1 DNA viral load in clinical specimens

**Figure 3:**
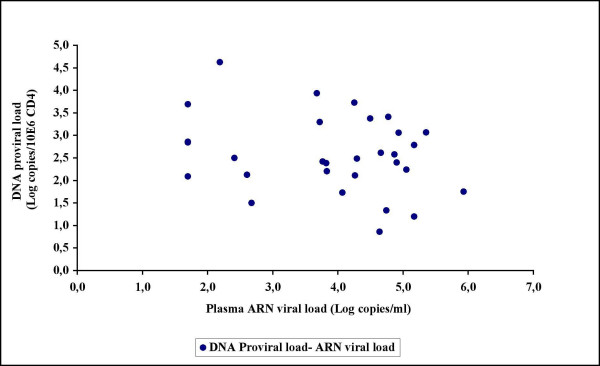
No correlation was found between DNA proviral load and plasma RNA viral load in thirty consecutive unselected patient samples received for RNA viral load determinations.

**Figure 4:**
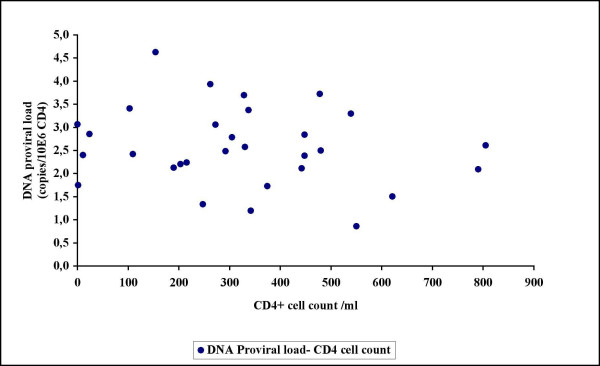
No correlation was found between DNA proviral load and CD4+ cell count in thirty consecutive unselected patient samples received for RNA viral load determinations.

**Figure 5:**
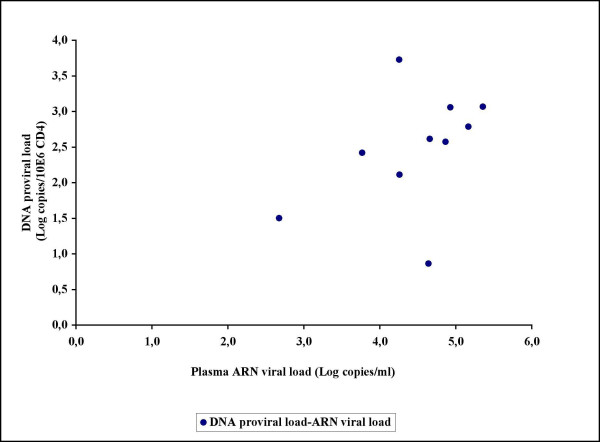
Correlative tendency between DNA relative proviral load and plasma RNA viral load in the naive patients but not statistically significant (p-value >0,05 by Pearson test)

In order to test our quantitative real-time PCR assay on DNA from patient CD4+ cell samples, the assay was first applied on thirty consecutive patient samples received for RNA viral load determinations regardless of whether or not they were receiving antiretroviral therapy. Of the 30 patients, 10 were treatment naive while 20 were antiretroviral-experienced adults (Table 2). If we put all patients (N = 30) together, no significant correlation was found between DNA relative proviral load and either plasma RNA viral load or CD4+ cell count (Figure 3 and Figure 4). The only observed correlative tendency was between DNA relative proviral load and plasma RNA viral load in the naive patients (Figure 5), but this was not statistically significant (p-value >0,05 by Pearson test). DNA proviral load varies widely among HIV-1 infected patients in the same or at different disease stages.

**Table 2:** Patient characteristics

	Naive group	Treated patients	STI patients
**Number**	10	20	15
**Genders**			
Males	5	8	8
Females	5	12	6
Transsexuals			1
Mean years old	42	42	43
**Geographic origin**			
Europeans	5	12	12
Other/Unknown	5	8	3
**Mean range follow up**			
Years^a^	4 (range 1 – 11) ^a^	7.5 (range 1 – 15)^a^	6 (range 2 – 7) ^a^
**CD4+ count**			
cells × 10^6^/l	Median 391 (range 109–805)	Median 338 (range 1 – 790)	Median 717 (range 216 – 1356)
<200 cells × 10^6^/l (Number) ^b^	1	6	0
**Plasma HIV-RNA**			
Log_10 _copies/ml	Median 4.8 (range 2.7 – 5.4)	Median 4.4 (range 1.7 – 5.9)	Median 3.2 (range 1.7 – 4.7) ^c^
<50 copies/ml (Number)^b^	0	5	3
**HIV1-DNA**			
Log_10 _copies/10^6 ^CD4 cells	Median 3.0 (range 2.6 – 3.7)	Median 3.3 (range 2.2 – 4.6)	Median 2.9 (range 2.1 – 4.0)
<5 copies/PCR (Number) ^b^	6	12	5

(a) Number of years between HIV-1 infection diagnosis and DNA proviral load testing.

(b) Number of patients with viral or proviral load under limit of quantification or with CD4+ count <200 cells 10^6^/l.

(c) Steady state viral load after STI.

**Figure 6:**
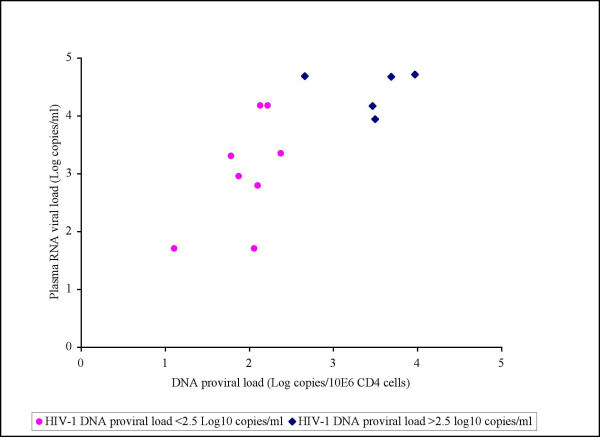
The assay was also applied on samples from 15 patients undergoing 2 years of STI. A statistically significant correlation was observed between DNA proviral load and RNA steady state viral load in STI patients. (p-value = 0,012, Pearson correlation test)

The assay was also applied on samples from fifteen patients undergoing 2 years of STI (Table 2). At the start of the STI, all patients were receiving at least a triple-therapy regimen including two nucleoside reverse transcriptase inhibitors (NRTIs) plus 1 or 2 PIs. All STI patients had received previous monotherapy or dual therapy before HAART with a mean total treatment time of five years. At the initiation of STI, 11 patients out of the 15 had an HIV-1 RNA viral load of less than 50 copies/ml; four had a baseline HIV-1 RNA viral load of less than 572 copies/ml (median of 2 log copies/ml) and the mean CD4+ cell count was 748 × 10^6 ^cells/l. Out of the 15 STI patients, seven had a steady state viral load less than 3.3 log copies/ml, including three patients with undetectable HIV-1 RNA viral load (*<*50 copies/ml), while eight patients had a high HIV-1 RNA viral load ranging from 3.9 to 4.7 log copies/ml. All seven patients with a low steady state plasma viral load showed a DNA proviral load under 2.5 log copies/10^6 ^CD4+ cells, including four patients with a proviral load under the limit of quantification (<5 DNA copies/PCR). Of eight patients with a high steady state plasma viral load (>3.9 log copies/ml), three showed a DNA proviral load under 2.5 log copies/10^6 ^CD4+ cells while five had a DNA proviral load above 2.5 log copies/10^6 ^CD4+ cells. A statistically significant correlation was observed between DNA proviral load and RNA steady state viral load in STI patients (Figure 6). The Pearson's coefficient of correlation was about 0,578 with an associated p-value of 0,012. When applying the nonparametric Spearman correlation method, the associated p-value was about 0,018. Proviral load and plasma viral load rebound were high in STI patients who took longer to achieve an undetectable plasma viral load under therapy.

## Discussion

A relative quantification assay of HIV-1 proviral DNA in purified CD4+ cells was developed on the LightCycler^® ^real-time PCR instrument expressed per 10^6 ^CD4+ cells as derived from estimated by the quantification of the β-globin gene. In healthy individuals, CD4+ cells represent approximately 30 to 50% of the PBMC and their fraction may be considerably lower and variable over time in HIV-infected patients. Theoretically, to improve the sensitivity and accuracy of an assay for low-copy-number samples, a larger amount of DNA should be added to the PCR reaction mix when purified from PBMCs than directly from CD4+ cells. High amounts of the cellular genome may lead to PCR inhibition. In addition, published data show low proviral loads, ranging from 1 to < 5 log_10 _HIV-1 DNA copies (median 2–3 log_10_) in 10^6 ^PBMC equivalents [14-17,19,20,22-24]. Methods quantifying the HIV-1 DNA proviral load in PBMC may therefore lead to undetectable proviral loads. However, in any case a direct comparison between DNA load in CD4+ cells and PBMCs could be useful as the CD4 isolation adds extra work (i.e. costs), which should be weighed against the clinical benefit. Sequence-specific hybridisation probes provide the most specific real-time analysis of amplified target sequences but the very high variability of HIV sequences within subtypes, even in HIV variants in a given patient led us to choose a sequence-independent detection with SYBR Green I. The ability of SYBR Green I to bind double-stranded DNA molecules with emission of a fluorescent signal allows the PCR reaction to be monitored. As SYBR Green I binds to all double-stranded DNA molecules, a melting temperature curve needs to be constructed to ensure the specificity of the signal. Thus specific primer selection and PCR optimisation is crucial to avoid primer dimer formation. We tested the influence of possible inhibitors in the DNA sample. A slight variation of the melting temperature (about 1°C) and the Cp values (about 1Cp) was observed, depending on the HIV-1 subtypes and on the type of initial sample (EDTA whole blood or culture supernatant). The specificity of the HIV-1 amplification was confirmed by the presence of the expected band size on the electrophoresis gel carried out with the PCR products from standards, reference strains, and patient samples. Direct sequencing of amplified products showed the expected HIV-1 sequences. No amplification was obtained with HIV-2 DNA. The ability of the assay to quantify HIV-1 Group M and the PCR efficiency were assessed on HIV-1 reference isolate subtypes A, B, C, and D. The results showed similar PCR efficiencies. Particular attention should be given to the quantification of the β-globin gene. We observed an inhibition with a high amount of the reference gene. Diluting (10-fold) DNA from patient samples resolves this problem. The lower limit of quantification is five HIV-1 DNA proviral copies per PCR and 1.5 pg of cellular DNA/μl (approximately five cells) for the human β-globin reference gene quantification. The dynamic range of HIV-1 DNA quantification is between 50 and 10^6 ^proviral copies per CD4 with coefficients of variability of the HIV-1 DNA copy number ranging from 3.1% for high provirus concentrations to 37.1% for low concentrations. Our method shows acceptable technical sensitivity and specificity. Quantification could be performed without the need to include a standard curve in every run by loading an external standard curve generated in a different run. Relative quantification was carried out by using the LightCycler^® ^Relative quantification Software (version 1.0). The result of HIV-1 proviral quantification was expressed as log_10 _number of DNA copies per 10^6 ^CD4+ cells. The crossing points that are calculated by the LightCycler^® ^Software are a function of the amplification efficiency. Efficiency differences have a major impact on the accuracy of initial amount calculation. The assay developed met one of the experimental prerequisites for PCRs, HIV-1 target gene and β-globin reference gene, i.e., having identical PCR efficiencies in sample, standard, and calibrator.

The assay was successfully tested on 30 consecutive unselected patient samples. No significant correlation was found between DNA relative proviral load and either plasma RNA viral load or CD4+ cell in this group, overall. Similar observations have been reported in the literature [9,24]. DNA proviral load varies widely among HIV-1 infected patients in the same or at different disease stages.

The assay was also applied on samples from 15 patients undergoing 2 years of STI. In fact, of the 15 STI patients, all seven patients with a low steady state plasma viral load showed a DNA proviral load under 2.5 log copies/10^6 ^CD4+ cells, including four patients with a proviral load under the limit of quantification (<5 DNA copies/PCR). Of the remaining eight patients presenting a high steady state plasma viral load (>3.9 log copies/ml), three showed a DNA proviral load under 2.5 log copies/10^6 ^CD4+ cells while five had a DNA proviral load above 2.5 log copies/10^6 ^CD4+ cells. We observed that in patients who needed a change in antiretroviral treatment to achieve an undetectable plasma viral load or who took longer to achieve an undetectable viral load under treatment, the proviral load was >2.5 log copies/PCR and the plasma viral load rebound was high. However, a large and prospective study is needed to confirm the value of this observation and particularly to assess the predictive value of proviral load in the setting of STI.

## Conclusion

We have developed a fast, sensitive and specific assay, which enables the monitoring of HIV-1 proviral load in CD4+ cells by LightCycler^® ^real-time PCR based on SYBR Green I quantification. This should enable us to evaluate prospectively the proviral load as a prognostic marker in therapy and in the evaluation of treatment interruptions.

## Competing interests

The author(s) declare that they have no competing interests.

## Authors' contributions

BKM conceived of the study and designed it together with PH and PG. BKM developed the HIV-1 DNA real-time PCR and performed the assay and sequencing reactions. PH assembled the clinical samples. ND did the CD4+ cell preparation and the all the viral culture work. BK and PG drafted the manuscript. JR and MB reviewed the manuscript. All authors contributed to the final version of the manuscript, read and approved it.

## Pre-publication history

The pre-publication history for this paper can be accessed here:


